# The relationship between emotional labor status and workplace violence among toll collectors

**DOI:** 10.1186/s40557-017-0193-9

**Published:** 2017-08-10

**Authors:** Yosub Joo, Jeongbae Rhie

**Affiliations:** 1 Department of Occupational and Environmental Medicine, Dankook University Hospital, Cheonan-si, South Korea; 2 Department of Occupational and Environmental Medicine, Dankook University College of Medicine, 119 Dandae-ro, Dongnam-gu, Cheonan-si, Chungcheongnam-do 330-715 South Korea

**Keywords:** Worker, Emotional stress, Workplace violence

## Abstract

**Background:**

This study aimed to identify the emotional labor and workplace violence status among toll collectors by assessing and comparing the same with that in workers in other service occupation. It also aimed to analyze the relationship between emotional labor and workplace violence.

**Methods:**

This study examined emotional labor and workplace violence status in 264 female toll collectors from August 20 to September 4, 2015. The emotional labor was assessed using the Korean Emotional Labor Scale (K-ELS), and a questionnaire was used to examine the presence or absence, and type and frequency of workplace violence experienced by the subjects. A linear regression analysis was also performed to analyze the relationship between workplace violence and emotional labor.

**Results:**

The scores on “emotional demanding and regulation (*p* < 0.001),” “overload and conflict in customer service (*p *= 0.005),” “emotional disharmony and hurt (*p* < 0.001),” and “organizational surveillance and monitoring (*p* < 0.001)” among the sub-categories of emotional labor were significantly high and indicated “at-risk” levels of emotional labor in those who experienced workplace violence, whereas they were “normal” of emotional labor in those who did not. Even after being adjusted in the linear regression analysis, the emotional labor scores for the above 4 sub-categories were still significantly high in those who experienced workplace violence. On comparing the present scores with 13 other service occupations, it was found that toll collectors had the highest level in “emotional disharmony and hurt,” “organizational surveillance and monitoring,” and “organizational supportive and protective system”.

**Conclusions:**

This study found that the toll collectors engaged in a high level of emotional labor. Additionally, there was a significant relationship between emotional labor and the experience of workplace violence among the toll collectors.

## Background

The current industrial structure is very different from that of the past. The roles of occupations that have been important in the past are diminishing or disappearing, and the industrial structure is changing rapidly owing to the advent of new occupations. The service industry occupies the largest portion of such changes [1].

According to data from the National Statistical Office, the total number of workers in South Korea was 26.14 million as of August 2015, of whom 5.83 million (22.3%) were service/sales workers [2]. In a study by Kim et al., (2014), an estimated 5.79 million workers are engaged in emotional labor in South Korea, and the work of about 8 million people is considered to entail interactions with “customers” [3].

As the number of workers in the service industry increases, there is an increasing interest in their health. However, the concepts of “exposure to hazardous substances” and “safety” issues applied to manufacturing workers are hardly applicable to service industry workers because work in the service industry is characterized by “emotional labor.”

The concept of emotional labor was first introduced by the American female sociologist, A. R. Hochschild, in her book titled “The managed heart” [4]. Hochschild defined emotional labor as “the management of the feeling to create a publicly observable facial and bodily display.” According to Hochschild, “this labor requires one to induce or suppress feelings in order to sustain the outward countenance that produces the proper state of mind in others.” The concept of emotional labor is distinguished from emotional management that is intended to maintain an amicable interpersonal relationship. Emotional labor has the following three characteristics: it involves face-to-face or voice-to-voice contact with customers, it requires workers to produce an emotional state in another person such as gratitude or fear, and it allows employers to exercise a certain degree of control over employees’ emotional activities through surveillance and monitoring [4]. Among the three characteristics of emotional labor proposed by Hochschild, the point that employers exercise a degree of control over employees’ emotional activities through monitoring is an important characteristic that distinguishes emotional laborers from the other workers. Emotional laborers’ efforts to manage facial and bodily displays and emotional expressions constitute a part of their labor, and when emotional laborers do not perform emotional display rules properly by regulating those emotions, they are subjected to regulations and disadvantages. Morris and Feldman, and Adelmann have also suggested similar concepts of emotional labor [5, 6].

Workplace violence is an important factor for increasing the degree of emotional labor among emotional laborers [7], and “client-initiated workplace violence,” in which “clients” become assailants, is separately categorized as workplace violence [8]. Emotional laborers are exposed to a lot of verbal and physical violence in their interactions with customers, and emotional labor is known to be highly correlated with workplace violence experience [9].

There have been recent studies on emotional labor of hotel workers [10, 11], department store workers [12] and public service workers [13, 14]. Most of the related domestic studies have used a 9-item, 5-point assessment scale developed by Morris and Feldman or a modified version of the assessment tool [5]. The Korean Emotional Labor Scale (K-ELS), which consists of 5 sub-categories and 24 items, was developed in 2014, by Chang et al., [7, 15], but not many studies have used the K-ELS.

There are about 7200 toll collectors at 1161 booths in South Korea. Toll collectors are expected to carry out a high degree of emotional labor due to the characteristics of work that basically involves facing “customers” and their job requires them to perform 20 tasks as per customer satisfaction manuals, in 7 s. According to a working conditions survey study on toll collectors, conducted in July 2015, the emotional labor and workplace violence status among toll collectors was not good [16].

In the present study, we intended to identify emotional labor status among toll collectors by assessing emotional labor among toll collectors using the K-ELS and comparing their scores with workers in other service occupations. We also attempted to identify workplace violence status to analyze the relationship between workplace violence and emotional labor.

## Methods

### Study subjects

This study was conducted with 300 female toll collectors working on the Seoul outer ring road, from August 20, 2015 to September 4, 2015, using a structured, self-administered questionnaire. A total of 264 subjects were finally analyzed for this study, excluding 36 female toll collectors who did not respond to the questionnaire.

### Variables

#### Emotional labor

Emotional labor was assessed using the K-ELS [15]. This tool comprises the following 5 sub-categories with their respective number of items: emotional demanding and regulation, 5 items; overload and conflict in customer service, 3 items; emotional disharmony and hurt, 6 items; organizational surveillance and monitoring, 3 items; and organizational supportive and protective system, 7 items; thus totaling 24 items. The assessment scores were calculated and converted into a maximum score of 100 points in accordance with the category-specific score conversion formula presented in the K-ELS guide, and they were classified as “normal” and “at-risk” levels based on the factor-specific reference values. To compare with the levels of emotional labor in other service workers, a comparison was made with the assessment results of 2221 workers from 13 different service occupations who were assessed during the development of the K-ELS [7].

$$ \mathrm{Converted}\  \mathrm{score}\ \mathrm{for}\ \mathrm{each}\  \mathrm{category}=\frac{\left(\mathrm{sum}\ \mathrm{of}\  \mathrm{score}\mathrm{s}\ \mathrm{on}\ \mathrm{each}\  \mathrm{item}\  \mathrm{in}\  \mathrm{the}\  \mathrm{respective}\  \mathrm{category}\hbox{-} \mathrm{number}\  \mathrm{of}\  \mathrm{item}\mathrm{s}\right)\times 100}{\left(\mathrm{the}\  \mathrm{highest}\  \mathrm{possible}\  \mathrm{total}\  \mathrm{score}\ \mathrm{for}\ \mathrm{the}\  \mathrm{respective}\  \mathrm{category}\hbox{-} \mathrm{number}\  \mathrm{of}\  \mathrm{item}\mathrm{s}\right)} $$

#### Workplace violence

We defined workplace violence as the experience of at least one of the 5 types of violence: unreasonable demand, insulting remark, swearing/verbal abuse, physical violence and sexual harassment. It was assessed in terms of whether the subjects had experienced the 5 types of violence at work and the average incidence of such experiences per month was also investigated. In addition, the types of perpetrators and the measures that workers could take in the event of workplace violence were also identified.

#### General characteristics

The years of service was classified into less than 5 years, 5 years–less than 10 years, and more than 10 years, and the employment status was classified into permanent worker and temporary worker. In terms of working hours, the average working hours per day and average working hours per week were examined.

### Statistical analysis

Means and standard deviations were computed for the converted score for each emotional labor scale category. Using the experience of workplace violence as an independent variable and the score on each emotional labor sub-category as a dependent variable, the t-test was performed to examine the relationship between the two variables. Further, using the experience of workplace violence as an independent variable and the emotional labor scale score as a dependent variable, a linear regression analysis was performed to calculate beta coefficients and standard errors. Adjustments were made for age, years of service, shift-work type, and working hours. The significance level of all statistics was set at *p* < 0.05. The Statistical Package for the Social Sciences (SPSS version 23.0) was used for the analysis.

## Results

The subjects of this study were 264 female toll collectors, and their mean age was 50.4 ± 4.6 years. Further, 180 subjects (68.2%) had more than 10 years of service, 61 subjects (23.1%) had more than 5 years to less than 10 years of service, and 23 subjects (8.7%) had less than 5 years of service. In terms of shift work type, 155 subjects (58.7%) were regular 3-shift workers, 94 (35.6%) were irregular 3-shift workers and 15 (5.7%) worked in other shifts. The mean working hours per day and per week were 8.0 ± 0.2 h and 40.3 ± 1.9 h, respectively. There were significant difference in the employment status, shift-work type and working hours according to the presence or absence of experience of workplace violence (Table 1).

**Table 1:** General characteristics of female toll collectors

	Toll collectors (*n* = 264)		Workplace Violence				*p*-value
			Yes (*n* = 235)		No (*n* = 29)		
	N	(%)	N	(%)	N	(%)	
Age							
30–39	7	(2.7)	6	(85.7)	1	(14.3)	0.575
40–49	96	(36.4)	88	(91.7)	8	(8.3)	
50–59	161	(61.0)	141	(87.6)	20	(12.4)	
Years of service							
Less than 5 years	23	(8.7)	20	(87.0)	3	(13.0)	0.715
5 to 10 years	61	(23.1)	56	(91.8)	5	(8.2)	
10 years or more	180	(68.2)	159	(88.3)	21	(11.7)	
Employment status							
Permanent worker	261	(98.9)	234	(89.7)	27	(10.3)	0.033
Temporary worker	3	(1.1)	1	(33.3)	2	(66.7)	
Shift-work type							
Regular 3 shifts	155	(58.7)	130	(83.9)	25	(16.1)	0.003
Irregular 3 shifts	94	(35.6)	92	(97.9)	2	(2.1)	
Others	15	(5.7)	13	(86.7)	2	(13.3)	
Working hours (per week)							
40 h or less	249	(94.3)	225	(90.4)	24	(9.6)	0.015
More than 40 h	15	(5.7)	10	(66.7)	5	(33.3)	

A total of 235 subjects (89.0%) responded that they had experienced workplace violence, whereas 29 (11.0%) responded that they had never experienced workplace violence. The average incidence of workplace violence experiences per month was 4.2. In terms of the type of workplace violence experienced, the average incidence of insulting remark experiences per month was the highest, at 4.1 times (Table 2).

**Table 2:** Incidence of workplace violence by workplace violence types in female toll collectors

	Number	(%)	Per month	
			Mean	±SD
Workplace violence				
Yes	235	(89.0)	4.2	±8.8
No	29	(11.0)		
Subcategories				
Unreasonable demand				
Yes	145	(54.9)	2.8	±3.1
No	119	(45.1)		
Insulting remark				
Yes	221	(83.7)	4.1	±4.7
No	43	(16.3)		
Swearing/Verbal abuse				
Yes	199	(75.4)	3.6	±5.0
No	65	(24.6)		
Physical violence				
Yes	12	(4.6)	2.0	±1.4
No	252	(95.5)		
Sexual harassment				
Yes	104	(39.4)	2.5	±3.5
No	160	(60.6)		

The perpetrators included customers in 209 cases (94.4%), managers/supervisors in 7 cases (3.2%), and co-workers in 2 cases (0.9%). When asked about the measures they could take when exposed to workplace violence, 77 subjects (32.8%) responded that they “can report to a security guard or supervisor about the incident,” 11 (4.7%) responded that they “can avoid the encounter when it is unmanageable,” 85 (36.2%) responded that they “can ask a colleague or supervisor to handle the encounter,” and 45 (19.1%) responded that they “can decline the unreasonable demand.” Further, 45 (19.1%) chose the response option “others” and the detailed response was “there is no action to take.”

The mean K-ELS scores for the subcategories “emotional demanding and regulation,” “overload and conflict in customer service,” “emotional disharmony and hurt,” “organizational surveillance and monitoring,” and “organizational supportive and protective system” were 78.86 ± 13.70 (normal: 0–76.66, at-risk: 76.67–100), 72.43 ± 17.22 (normal: 0–72.21, at-risk: 72.22–100), 73.53 ± 17.00 (normal: 0–63.88, at-risk: 63.89–100), 63.38 ± 20.45 (normal: 0–49.99, at-risk: 50.00–100) and 59.97 ± 18.27 (normal: 0–45.23, at-risk: 45.24–100), respectively. All indicated “at-risk” level scores (Table 3).

**Table 3:** Mean scores on the Korean Emotional Labor Scale and respective mean scores according to the presence of workplace violence in female toll collectors

	Toll collectors (*n* = 264)		Workplace Violence				
			Yes (*n* = 235)		No (*n* = 29)		*p*-value
	Mean	±SD	Mean	±SD	Mean	±SD	
Emotional demanding and regulation	78.86	±13.70	80.03	±13.48	69.43	±11.89	<0.001
Overload and conflict in customer service	72.43	±17.22	73.57	±17.27	63.22	±13.96	0.002
Emotional disharmony and hurt	73.53	±17.00	75.20	±16.32	59.96	±16.49	<0.001
Organizational surveillance and monitoring	63.38	±20.45	65.39	±20.15	47.13	±15.04	<0.001
Organizational supportive and protective system	59.97	±18.27	60.61	±18.66	54.84	±13.92	0.109

The scores for “emotional demanding and regulation” (*p* < 0.001), “overload and conflict in customer service” (*p* = 0.002), “emotional disharmony and hurt” (*p* < 0.001) and “organizational surveillance and monitoring” (*p* < 0.001) among the subcategories of emotional labor indicated normal scores in subjects who did not experience workplace violence, and these scores were significantly higher, and at the at-risk level for those who did. For “organizational supportive and protective system,” there was no significant difference in the emotional labor scale score according to the presence or absence of experience of workplace violence, and the respective scores were high and indicated at-risk levels of emotional labor in those who experienced workplace violence (60.61 ± 18.66) and those who did not (54.84 ± 13.92) (Table 3).

When adjusted for age, years of service, shift-work type, and working hours in the linear regression analysis, the emotional labor scale scores for the subcategories “emotional demanding and regulation” (β = 9.27, *p* < 0.001), “overload and conflict in customer service” (β = 9.54, *p* = 0.005), “emotional disharmony and hurt” (β = 13.36, *p* < 0.001), and “organizational surveillance and monitoring” (β = 15.53, *p* < 0.001) were significantly higher in those who experienced workplace violence as compared to those in subjects who did not. However, there was no significant difference for “organizational supportive and protective system” (Table 4).

**Table 4:** Beta coefficients and standard errors for the Korean Emotional Labor Scale according to workplace violence in female toll collectors

	Crude				Adjusted^a^		
	β	SE	*p*-value		β	SE	*p*-value
Emotional demanding and regulation	10.60	2.62	<0.001		9.27	2.63	<0.001
Overload and conflict in customer service	10.35	3.34	0.002		9.54	3.39	0.005
Emotional disharmony and hurt	15.24	3.22	<0.001		13.36	3.23	<0.001
Organizational surveillance and monitoring	18.26	3.87	<0.001		15.53	3.84	<0.001
Organizational supportive and protective system	5.76	3.58	0.109		4.87	3.62	0.180

^a^β coefficients and standard errors were estimated using a linear regression analysis adjusted for age, years of service, shift-work type, and working hours (per week)

On comparing the present scores with those of the emotional labor assessments of 2221 workers in 13 other service occupations, it was found that toll collectors had the 7th highest level of emotional labor in “emotional demanding and regulation,” the 3rd highest level in “overload and conflict in customer service,” and the highest level in “emotional disharmony and hurt,” “organizational surveillance and monitoring,” and “organizational supportive and protective system” (Table 5).

**Table 5:** Comparison of scores on the Korean Emotional Labor Scale between toll collectors and workers in other service occupations

	Emotional demanding and regulation		Overload and conflict in customer service		Emotional disharmony and hurt		Organizational surveillance and monitoring		Organizational supportive and protective system	
Ranking	Type of occupation	Score^a^	Type of occupation	Score^a^	Type of occupation	Score^a^	Type of occupation	Score^a^	Type of occupation	Score^a^
1	Call center operator	17.45	Call center operator	10.04	Toll collector	19.23	Toll collector	8.70	Toll collector	19.59
2	Teller	17.40	After-sales service worker	9.56	Call center operator	17.98	After-sales service worker	8.49	After-sales service worker	19.18
3	Department store salesperson	17.19	Toll collector	9.51	Drivers of public transportation	17.81	Teller	8.32	Hospital receptionist	17.22
4	Hotel staff	17.12	Teller	9.50	Teller	17.59	Call center operator	8.03	Public office receptionist	17.11
5	After-sales service worker	16.97	Hotel staff	9.46	After-sales service worker	17.59	Hotel staff	7.86	Call center operator	17.09
6	Daycare worker	16.88	Nurse	9.40	Hotel staff	17.21	Flight attendant	7.74	Department store salesperson	16.88
7	Toll collector	16.83	Department store salesperson	9.29	Case worker	17.01	Fire fighter	7.72	Case worker	16.73
8	Nurse	16.77	Drivers of public transportation	9.27	Nurse	16.87	Nurse	7.57	Hotel staff	16.58
9	Hospital receptionist	16.64	Hospital receptionist	9.16	Public office receptionist	16.64	Department store salesperson	7.51	Nurse	16.54
10	Drivers of public transportation	16.60	Public office receptionist	8.92	Hospital receptionist	16.60	Drivers of public transportation	7.40	Fire fighter	16.54
11	Public office receptionist	16.30	Fire fighter	8.90	Department store salesperson	16.52	Public office receptionist	7.33	Drivers of public transportation	16.52
12	Fire fighter	16.07	Daycare worker	8.54	Flight attendant	16.30	Hospital receptionist	7.02	Flight attendant	16.50
13	Case worker	15.64	Case worker	8.52	Fire fighter	16.16	Daycare worker	6.61	Teller	15.89
14	Flight attendant	15.05	Flight attendant	8.50	Daycare worker	16.09	Case worker	6.45	Daycare worker	15.74

^a^Mean scores on the Korean Emotional Labor Scale (scores have not been converted to the 100-point scale)

## Discussion

The results of the present study showed that workplace violence increased the intensity of emotional labor. In particular, workplace violence was found to significantly increase the emotional labor scale scores for the subcategories “emotional demanding and regulation” (β = 9.27, *p* < 0.001), “overload and conflict in customer service” (β = 9.54, *p* = 0.005), “emotional disharmony and hurt” (β = 13.36, *p* < 0.001) and “organizational surveillance and monitoring” (β = 15.53, *p* < 0.001). In the present study, the emotional labor and workplace violence status among toll collectors were found to be serious. In addition, the toll collectors showed “at-risk” level scores in all 5 emotional labor sub-categories, and performed a higher intensity of emotional labor than did workers in other service occupations. The results of our comparison with the emotional labor scores of 2221 workers from 13 other service occupations revealed that the toll collectors had the highest emotional labor scores for the subcategories “emotional disharmony and hurt,” “organizational surveillance and monitoring,” and “organizational supportive and protective system” [7].

A previous working conditions survey on toll collectors also used the K-ELS [16]. When compared with the results of the present study, there were differences in the subcategories indicating “at-risk” level scores. Unlike the results of the present study showing at-risk levels of emotional labor in all 5 categories, the previous study showed “at-risk” level scores on 3 categories, “overload and conflict in customer service,” “emotional disharmony and hurt,” and “organizational surveillance and monitoring.” However, the previous study was a nationwide survey, and the toll collectors working in similar areas to the subjects of the present study had higher scores for all categories. This is considered to be due to the fact that toll gates are concentrated on the Seoul Outer Ring Road and the number of passing vehicles is much higher than that on roads in other areas. In addition, the previous study assessed workplace violence using the Korean Workplace Violence Scale (K-WVS), and found that the experience of psychological and sexual violence from customer was at the “at-risk” level. Although the present study did not use the K-WVS, the results showed that most of the workplace violence was caused by “customers” (94.4%), which was consistent with the results of the previous study.

The International Labor Organization (ILO) categorizes workplace violence into the following 3 types: “external intruder workplace violence” by criminal intruders (or Type 1), “client or customer perpetrators of workplace violence” by dissatisfied clients, recipients or patients of a service (or Type 2), and “internal perpetrators of workplace violence” by co-workers or supervisor/managers (or Type 3) [17]. Such violence includes threat, physical violence, psychological violence, and verbal violence.

In the present study, 89.0% of the toll collectors experienced workplace violence, and the proportion of those who experienced verbal violence such as insulting remarks (83.7%) and swearing/verbal abuse (75.4%) was greater than that of those who experienced other forms of violence. For those who experienced workplace violence, the scores for the 4 subcategories of emotional labor scale were significantly high and they were at the “at-risk” level for emotional labor, which suggests that preventing workplace violence can significantly reduce the intensity of emotional labor among workers.

The 5th subcategory of emotional labor, “organizational supportive and protective system,” assesses the presence of a system to support workers when problems arise during emotional labor and the degree of co-workers’ support. Thus, it is a measure of whether the workplace and its employees are properly protected. The scores for other 4 subcategories were in the “at-risk” range for those who experienced workplace violence and in the “normal” range for those who did not. However, the score for the category “organizational supportive and protective system” was high and in the “at-risk” range even for those who did not experience workplace violence. This means that, regardless of the experience of workplace violence, the workers feel a lack of “organizational supportive and protective system.” It is evident that they work under the anxiety that they may not be protected by the organization even if workplace violence does not occur.

This can be confirmed by the results of our survey on the measures that can be taken in the event of workplace violence. Only 32.8% of the subjects responded that they “can report to a security guard or supervisor about the incident,” 19.1% responded that they “can decline unreasonable demand,” and only 4.5% responded that they “can avoid the encounter when it is unmanageable.”

Toll collectors are not simply involved in collecting the toll fee. They are also expected to respond to customers asking for directions, and if this causes any delay, they have to listen to the complaints and verbal abuse of customers who are waiting in the back. Customers do not just stop here, and may harass them through filing civil complaints. If such civil complaints occur, the tool collectors may face disciplinary action or may have their pay cut. They are thus forced to accept the verbal abuse or unreasonable demands of customers. Even if they suffer from depression due to the high intensity of emotional labor, they are often afraid of being dismissed, and they have to hide their feeling or do not receive proper treatment or counseling. Unlike situations in other workplaces, any perpetrator of verbal abuse at tollgates can pass by in his/her vehicle. In addition, toll collectors are prohibited from carrying mobile phones while on duty, and thus they cannot take recordings and follow-up actions [18]. As such, workplace systems, by which toll collectors exposed to workplace can be protected, are very vulnerable, suggesting that improving emotional labor-related protection systems in the workplace should be prioritized.

Emotional labor has both positive and negative effects on workers. Grandey suggested that emotional labor affects health, and individuals and organizations in the following three stages: situational cues, emotional regulation process, and long-term consequences [19]. Positive effects related to emotional labor include smiling expressions affecting blood circulation and neurotransmitters to induce pleasant internal emotions [20], and the sense of accomplishment and sense of self-efficacy derived from fulfilling job demands [21].

The negative effects of emotional labor include both physical and mental effects, and several studies have been conducted in this regard. As the level of emotional labor increases, so does the level of job [22]. Stress can act as a causal and exacerbating factor for cerebral and cardiovascular diseases [23], and can further increase the risk of cerebral and cardiovascular diseases in conjunction with shift work [24]. In addition, it is known that emotional labor workers are at a higher risk of developing mental illnesses such as depression [25, 26] and are at a significantly higher risk of developing musculoskeletal disorders [14]. There is also a previous study showing that emotional labor was associated with dysmenorrhea in female workers [27]. It is also known that those who experienced workplace violence are at a significantly high risk of subjective dysthesia, depressive symptoms, and presenteeism and absenteeism [3].

According to a report analyzing occupational accident claims, 502 occupational accident claims for mental illness were submitted from 2005 to 2012, of which, 40 claims (8.2%) were related to emotional labor workers. Further, of the occupational accident claims for mental illness, 31.1% were submitted by those who had experienced workplace violence [3].

To minimize the negative physical and mental health effects of emotional labor, personal, legal, and institutional measures for the protection of emotional labor workers are needed. At the individual level, workers must be aware of whether his/her work is applicable to emotional labor. For this, publicity and education about the concept, prevention, and countermeasures of emotional labor in the workplace are needed. The Occupational Safety and Health Act, Article 5 states the employers’ obligations related to employees’ mental stress, and the Enforcement Rules to Occupational Safety and Health Standards, Article 669 stipulates the employers’ obligations to prevent health problems caused by job stress. In the past, there were many occupations that were classified as exempted from the law, but with changes in the industrial structure, the application of the law expanded to a considerable number of service industries which were previously exempted from the law. However, several occupations are still exempted under the Occupational Safety and Health Act, Article 31 (Health and Safety Education). Considering that a considerable number of workers in those exempted occupations are emotional laborers working in interactions with customers, safety and health education about emotional labor and the prevention of and countermeasures against workplace violence should be provided to them through expanded application of related laws to these occupations.

The most urgent part of legal and institutional measures is about violence by “third parties.” As found in the present study, most of the perpetrators of workplace violence were “customers,” but domestic laws and regulations do not have provisions on violence by third parties and there is no related obligation and liability of employers. Meanwhile, in Europe, such as in the UK, Germany, and Belgium, violence by third parties is specified separately and related obligations and liabilities of employers are imposed by the law [28–30].

Considering the results of this study showing that the score for the category “organizational support and supportive system” indicated scores at the “at-risk” level regardless of the experience of workplace violence, it is necessary to establish a step-by-step and systematic protection system for emotional laborers. The Canada Occupational Safety and Health Regulations sets out a 7-step approach to address workplace violence [31], and the guide on related legal regulations also presents 6 steps for workplace violence prevention [32].

The protection of workers from workplace violence by third parties and the establishment of protection system related to emotional labor can be achieved through the provision of legal and institutional devices. However, a report published in 2008 by the Health and Safety Executive (HSE) in the UK pointed out that there are limitations to the establishment of laws and institutions concerning emotional labor [33]. In this report, it is stated that the relationship between the characteristics of emotional labor and associated health impact may be context-specific, and thus, presenting overall management of emotional labor across all occupations should be avoided. In addition, it was pointed out that because emotional labor has both positive and negative aspects, approaches to reduce or control emotional labor may not be appropriate. Therefore, it was concluded that dealing with emotional labor together with job demands may be more effective than dealing with emotional labor alone.

As such, despite the fact that the level of emotional labor among emotional labor workers is very high, there are many difficulties in preparing measures to reduce the associated negative impact on workers. Because each service has different characteristics and related job demands vary, objective indicators of job demands related to emotional labor should be established along with assessments of emotional labor. In addition, guidelines should be developed for each of the 5 sub-categories of the K-ELS, and institutional frameworks that specify related obligations of employers and employees should be provided.

The present study has several limitations. First, the difference in the workload of the toll collectors was not considered. For toll collectors, the number of customers they meet may vary due to the difference in traffic volume depending on where they work, which can change the intensity of emotional labor. Second, there was a lack of further investigation of working conditions that could affect emotional labor among the toll collectors. The job of toll collectors is characterized by high exposure to exhaust gases, inability to go to the toilet when required, and the expectation to perform asymmetrical and repetitive movements. It is regrettable that the working environment factors were not assessed in the present study, with no evaluation of respiratory, urinary, and musculoskeletal diseases, and no analysis of the effects of those factors on emotional labor.

However, despite these limitations, our study is significant and has some strengths. This study is significant in that this study used a standardized instrument, the K-ELS, and reconfirmed that the emotional labor status among toll collectors was serious. In addition, the present study examined the relationship between workplace violence status and the sub-categories of emotional labor and thus presented direction for the improvement of the working environment of toll collectors. Overall assessments of workers in other service occupations using the K-ELS are needed in the future, and the assessment results of emotional laborers will play an important role in establishing related legal and institutional frameworks.

## Conclusions

The toll collectors showed “at-risk” level scores in all 5 emotional labor sub-categories, and performed a higher intensity of emotional labor than did workers in other service occupations. For those who experienced workplace violence, the scores for the 4 subcategories of emotional labor scale were significantly high and they were at the “at-risk” level for emotional labor, which suggests that preventing workplace violence can significantly reduce the intensity of emotional labor among workers. To protect workers in the service industry from emotional labor and workplace violence, a step-by-step and systematic protection system in the workplace and legal and institutional measures should be instituted.

## Abbreviations

HSE: Health and Safety Executive
ILO: The International Labor Organization
K-ELS: Korean Emotional Labor Scale
K-WVS: Korean Workplace Violence Scale
